# Functional Identification and Characterization of the Diuretic Hormone 31 (DH31) Signaling System in the Green Shore Crab, *Carcinus maenas*

**DOI:** 10.3389/fnins.2018.00454

**Published:** 2018-07-04

**Authors:** Jodi Alexander, Andrew Oliphant, David C. Wilcockson, Simon G. Webster

**Affiliations:** ^1^Brambell Laboratories, School of Biological Sciences, Bangor University, Bangor, United Kingdom; ^2^Institute of Biological, Environmental and Rural Sciences, Aberystwyth University, Aberystwyth, United Kingdom

**Keywords:** *Carcinus maenas*, calcitonin-like diuretic hormone 31, G protein-coupled receptor, neuroanatomy, mRNA and peptide expression, physiological roles

## Abstract

The functional characterization of crustacean neuropeptides and their cognate receptors has not kept pace with the recent advances in sequence determination and, therefore, our understanding of the physiological roles of neuropeptides in this important arthropod sub-phylum is rather limited. We identified a candidate receptor-ligand pairing for diuretic hormone 31 (DH31) in a neural transcriptome of the crab, *Carcinus maenas*. In insects, DH31 plays species -specific but central roles in many facets of physiology, including fluid secretion, myoactivity, and gut peristalsis but little is known concerning its functions in crustaceans. The *C. maenas* DH31 transcript codes for a 147 amino acid prepropeptide, and a single receptor transcript translates to a secretin-like (Class B1) G protein-coupled receptor (GPCR). We used an *in vitro* aequorin luminescence Ca^2+^ mobilization assay to demonstrate that this candidate DH31R is activated by*Carcinus* and insect DH31s in a dose-dependent manner (EC_50_ 15–30 nM). Whole mount immunohistochemical and *in situ* hybridization localization revealed extensive DH31 expressing neurons throughout the central nervous system, most notably in the abdominal ganglion where large, unpaired cells give rise to medial nerves, which terminate in extensive DH31 immunopositive dendritic fields intimately associated with oesophageal musculature. This system constitutes a large and hitherto undescribed neurohemal area adjacent to key muscle groups associated with the gastric system. DH31 expressing neurons were also seen in the cardiac, commissural, oesophageal, and stomatogastric ganglia and intense labeling was seen in dendrites innervating fore- and hindgut musculature but not with limb muscles. These labeling patterns, together with measurement of DH31R mRNA in the heart and hindgut, prompted us test the effects of DH31 on semi-isolated heart preparations. Cardiac superfusion with peptide evoked increased heart rates (10–100 nM). The neuroanatomical distribution of DH31 and its receptor transcripts, particularly that associated with gastric and cardiac musculature, coupled with the cardio- acceleratory effects of the peptide implicate this peptide in key myoactive roles, likely related to rhythmic coordination.

## Introduction

In recent years, *de novo* assembly and data mining of transcriptomes and genomes of arthropods has revealed a wonderfully rich and diverse collection of neuropeptide signaling molecules, and their putative (mainly G protein- coupled) receptors (GPCRs). Many of these are common to both insects and crustaceans- perhaps unsurprisingly, given the consensus that all arthropod lineages arose from a monophyletic ancestor (Cook et al., 2001; Regier et al., 2010). An inevitable consequence of the rapid expansion of arthropod neuropeptidomes is that we still know remarkably little regarding the functions of most of these neuropeptides, and this is particularly striking for crustaceans; *in silico* and *de novo* assembly of transcriptomic and genomic data has led to the discovery of an impressive number of deduced neuropeptide homologs. Examples are listed in Christie and Chi (2015), and of particular relevance are those in which putative GPCR/ligand pairings have also been suggested from *de novo* transcriptome assemblies, for example *Calanus finmarchicus* and *Homarus americanus* (Christie et al., 2013, 2017) or genome annotations, *Daphnia pulex* (Colbourne et al., 2011).

As alluded to earlier, a striking feature of these assembled transcriptomes is the evident conservation of structures of many of the peptide groups, and obvious relatedness to insect neuropeptides, however, due to the genetic intractability of most crustaceans (and until recently a lack of potentially useful, genome sequenced models), relatively slow progress has been made regarding identification of the biologically relevant functions of crustacean peptides. During the passing of 400 million years of divergent arthropod evolution, commonality in structure across the phylum cannot be assumed to reflect current function.

To begin to address the issue of peptide functionality, we reasoned that a potentially useful approach would be one involving “reverse endocrinology”- to identify peptide receptor signaling pathways, by functional confirmation of putative GPCR/ligand pairings, and to couple this with identification and quantification of tissue specific receptor and ligand expression with the eventual aim of determination of functions.

Following the assembly of *de novo*, molt stage specific neural transcriptomes of the green shore crab, *Carcinus maenas* (Oliphant et al., unpublished), a candidate ligand and putative GPCR pairing was that of the calcitonin-like diuretic hormone (CLDH or diuretic hormone, DH31) signaling system. DH31 is considered to be the homolog of vertebrate calcitonin since it bears some (rather limited) sequence identity, including the C-terminal GP-amide to this peptide (review: Schooley et al., 2012). Calcitonin-type molecules are undoubtedly evolutionarily ancient, since deuterostomian type calcitonins (which contain two conserved Cys residues) are present in lophotrochozoans, together with DH31-like peptides (Conzelmann et al., 2013), and the former (calcitonins A, B) are present in many, but not all insects (Veenstra, 2014). DH31was first reported as an insect neuropeptide from *Diploptera punctata* that stimulated cAMP production by MT of *Schistocerca americana* (Furuya et al., 2000). In insects DH31 increases fluid secretion by the MTs (Furuya et al., 2000) and stimulates natriuresis in *Anopheles gambiae* (Coast et al., 2005), but in *Rhodnius prolixus*, it is not only active in stimulating MT fluid transport, but is a potent myoactive peptide, causing cardioacceleration and increased hindgut contractility (Te Brugge et al., 2008). In *Drosophila*, DH31 expressing interendocrine cells in the midgut modulate peristalsis (LaJeunesse et al., 2010). Intriguingly, DH31 and the PDFR are involved in temperature preference rhythms (Goda et al., 2016) and the DH31 receptor (DH31R) is expressed in clock cells in *Drosophila* (Goda et al., 2018). Furthermore, PDF signaling in the dorsal clock neurons which express DH31 seems to be involved in wake-promoting signals in *Drosophila*, (Kunst et al., 2014), suggesting important roles in the circadian clock. Taken together, these studies undoubtedly reflect complex and central roles for the DH31 signaling system in many aspects of behavior and physiology in insects.

In crustaceans DH31 seems to be common, probably ubiquitous- all the transcriptomes (indeed sequences from a large number of arthropod species) contain highly conserved, easily recognizable DH31 peptide (and to a certain extent, prepropeptide) sequences (review, Zandawala, 2012). However, to date, the only known action of DH31 in crustaceans is as a potent cardioaccelerator in *H. americanus* and it is likely an intrinsic neuromodulator of the cardiac neuromuscular system in this animal (Christie et al., 2010).

With regard to DH31 receptors, a number of these have been predicted in insects from protein BLAST searches (see Caers et al., 2012 for list), but relatively few have been functionally confirmed using heterologous cell-based reporting systems. Examples are *Drosophila* (Johnson et al., 2005), *Rhodnius prolixus* (Zandawala et al., 2013), *Bombyx mori* (Iga and Kataoka, 2015), or by RNAi and immunochemical visualization in *Aedes aegypti* (Kwon and Pietrantonio, 2013). Although several putative crustacean neuropeptide receptors, including that of DH31 have been proposed from *de novo* transcriptome assemblies in *C. finmarchicus* and *H. americanus* (Christie et al., 2013, 2017), to date very few of these have been functionally confirmed in crustaceans, which is a mandatory step in unequivocal receptor identification. To date the only ones thus identified are the red pigment concentrating receptor (RPCHR) in *D. pulex* (Marco et al., 2017), RPCHR and the related CRZR that we have recently functionally confirmed in *C. maenas* (Alexander et al., 2018).

To begin to address questions regarding the functionality of the DH31 ligand and receptor signaling system, we have functionally identified the (GPCR) receptor in *C. maenas*, and have detailed tissue and molt-stage specific expression patterns for both peptide and receptor. Coupling these studies with a detailed description of the neuroanatomy of immunoreactive structures in the central and peripheral nervous tissues, together with bioassays, we propose the working hypothesis that DH31 is involved in a variety of (myo) activities, most likely related to rhythmic behaviors and ecdysis, which strongly suggest a central role in crustacean physiology.

## Materials and Methods

### Animals and Tissue Collection

Specimens of mature green shore crab, *C*. *maenas* were collected using baited traps from the Menai strait, United Kingdom. Crabs were maintained in a recirculating seawater system at ambient temperature and photoperiod, and were fed with fish. Tissues from molt-staged animals (Drach and Tchernigovtzeff, 1967) were dissected, following anesthesia on ice, and processed either for immunohistochemistry (IHC), with Stephanini’s fixative (Stephanini et al., 1967), (overnight, 4°C), or for *in*-*situ* hybridization (ISH), 4% PFA in PBS, overnight at 4°C. For RNA extractions and peptide quantification, tissues were dissected, immediately frozen in liquid nitrogen, and stored at -80°C.

### Transcriptome Data Mining

Transcriptome sequencing of neural tissue was performed as described elsewhere (Oliphant et al., unpublished). Transcriptomes were mined for contigs putatively encoding neuropeptides and GPCRs using tBLASTn local searches in BioEdit software (Hall, 1999). Neuropeptide and GPCR protein sequences used as search terms using sequence data from *Tribolium castaneum* and *Drosophila melanogaster* were taken from the NCBI database Contigs mined as putative neuropeptide receptor sequences were translated using ExPASy translate^1^ (Artimo et al., 2012), submitted to tBLASTn searches against the NCBI database, and transmembrane domains predicted using TMHMM server v2.0^2^(Sonnhammer et al., 1998).

### Quantitative RT-PCR

Tissue and molt stage specific transcript expression of the DH31 receptor (DH31R) and DH31 was performed using Taqman MGB hydrolysis probes as previously described (Hoelters et al., 2016). Standard cRNAs were prepared by *in vitro* transcription (Megashortscript, Ambion) of PCR products generated using gene specific primers flanked by T7 phage promoter sequences. cRNAs were gel purified (10% 6M urea PAGE) and correctly sized transcripts excised and eluted. Copy number of quantified cRNA was calculated using Avogadro’s constant and standard curves were run in the range 10^8^–10^2^ copies. Standards and 1 μg DNase treated (Turbo DNase, Ambion) samples were reverse transcribed using anchored oligo dT primers and Tetro cDNA kits (Bioline United Kingdom) according to the manufacturer’s instructions. Duplex qPCR reactions (10 μl) which simultaneously amplified target and reference genes were performed in triplicate using Sensifast Probe II reagents (Bioline) and run on an Applied Biosystems QuantStudio 12-Flex machine. Data were expressed as copies of cDNA target, normalized to the geometric mean of the stably expressed *elongation factor-1* and *ubiquitin*-*conjugating enzyme E2* L3 (Oliphant et al., unpublished). Primer sequences are shown in Supplementary Table S1).

### DH31 Receptor Assays

#### Isolation and Cloning of DH31R

Neural tissue transcriptome mining revealed single candidates encoding a putative DH31, and one encoding a GPCR with high amino acid sequence identity to those of insect GPCRs encoding putative DH31Rs. To prepare PCR products for cloning, total RNA was extracted from neural tissues (TRIzol, Invitrogen), followed by gDNA removal with *DNase1* (TURBO DNA-free, Ambion) and further separation of mRNA using Dynabeads Oligo (dT)_25_ (Dynal, Oslo, Norway). cDNA was prepared using a random hexamer/oligo (dT)_18_ mix and Tetro cDNA synthesis kit (Bioline, United Kingdom) according to the manufacturer’s instructions. Gene specific primers for DH31R were designed to span the ORF (Supplementary Table S1), and the target amplified using Phusion^®^ High Fidelity DNA polymerase (New England BioLabs, Ipswich, MA, United States) in 25 μl reactions. Conditions were: 98°C 7min, 35 cycles of 98°C 30 s, 65–72°C 30 s, 72°C 1 min 45 s, final extension 72°C 10min. PCR products were purified on 2% agarose gels, and bands of the correct size excised and purified (Gel Extract Mini Kit, 5Prime, Hamburg). Correctly sized PCR products were directionally cloned into pcDNA^TM^ 3.1 D/V5-His-TOPO plasmids and the recombinant vector transformed into TOP 10 competent cells (Invitrogen). Positive clones were cultured overnight in LB with 100 μg ml^-1^ ampicillin, plasmids extracted (FastPlasmid Mini Kit, 5Prime), sequenced (MWG Eurofins, Ebersberg, Germany), and analyzed using Geneious V 9.1.8 (Kearse et al., 2012).

#### Cell Culture and Receptor Assays

Chinese hamster ovary cells (CHO-K1) expressing apoaequorin (Perkin Elmer, Boston, MA, United States) and either the Gα16 or G*_q_* subunit were routinely cultured in Dulbecco’s Modified Eagles medium (DMEM) F-12 Nutrient Mixture GlutaMax (Gibco^®^) with 10% fetal bovine serum at 37°C, 5% CO_2_ and grown to 60% confluence in T75 flasks before transfection. Medium for transfection consisted of 800 μl Opti-MEM (Gibco), 10 μl recombinant vector, 30 μl FugeneHD (Promega) which was incubated for 10 min RT, added to 5 ml fresh culture medium, then added to the cells and incubated for 24 h. At this time, 10 ml of culture medium was added to the flasks and cells were incubated overnight.

On the day of the assay, cells were detached (5 ml 0.2% EDTA in PBS, 12 min), washed in clear DMEM/F12 with glutamine and 50 mM HEPES, centrifuged (260 *g*, 5 min) and resuspended in sterile BSA medium (DMEM/F-12, 50 mM HEPES, 0.1% BSA) to give a cell concentration of 5 × 10^6^ cells ml^-1^. Coelenterazine *h* (Invitrogen) was added to the cells to give a final concentration of 5 μM and incubated in the dark (4 h) with gentle rocking. Cells were then diluted 10-fold prior to assay.

Peptides used in the Ca^2+^ evoked luminescence assay were: synthetic C. *maenas* DH31 (Genecust Dudelange, Luxembourg), C. *maenas* pigment dispersing hormone-1 (PDH-1, Liverpool University), *Diploptera punctata* DH31, *Tribolium castaneum* DH31 (D. Schooley, Nevada), Alexa 488-*Drosophila melanogaster* DH31 (J. Dow, Glasgow), *Rhodnius prolixus* CRF-like DH (I. Orchard, Toronto). Amino acid sequences are shown in Supplementary Table S2. Peptides were reconstituted in 30% acetonitrile, aliquoted and dried by vacuum centrifugation and subsequently redissolved in BSA medium. Peptides were dispensed into quadruplicate wells of white 96 well plates (OptiPlate, Perkin Elmer). Cell suspensions were gently stirred and 50 μl amounts were injected into each well using a Mithras LB940 microplate reader (Berthold Technologies, Bad-Wildbad, Germany). Ca^2+^ evoked luminescence was recorded for 40 s, followed by cell lysis (injection of 0.3% Triton-X 100 in BSA medium), and light emission recorded for a further 10 s. BSA medium was used for blank measurements (six replicates per plate), and mock transfections with empty vectors for negative controls. Data reduction and analysis was done using MikroWin v5.18 (Mikrotek Laborsysteme, GmBH) and SigmaPlot v.13 (Systat Software Inc.). Receptor responses were normalized against total Ca^2+^ luminescence.

### Immunohistochemistry and *in Situ* Hybridization

An antiserum was raised in rabbits using synthetic *C*. *maenas* DH31, coupled to bovine thyroglobulin and the resulting antiserum was affinity purified with immobilized DH31 ligand (Davids Biotechnologie, Ulm, Germany). Specificity of the antiserum was evaluated by (a) preabsorbtion of 10-1-fold molar excess of DH31 (Supplementary Figure S1), (b) using preimmune serum and (c) HPLC of nervous system extracts followed by TR-FIA. For some preparations, an Alexa 594 labeled antiserum was used. This was prepared by conjugating *N*-hydroxysuccinamide-labeled Alexa 594 to milligram amounts of affinity purified DH31 IgG using a microscale labeling kit (Invitrogen, Thermo Fisher Scientific).

Fixed nervous systems were processed for IHC as detailed in Webster et al. (2013). Primary antiserum concentrations were 1:6000–1:12000. Secondary antiserum dilution (Alexa Fluor 488 goat anti-rabbit, Invitrogen, Thermo Fisher Scientific) was 1:750. For double labeling experiments, preparations were co-incubated with Alexa 594 DH31 (1:2000) and *Carcinus* anti-Burs IgG [raised in guinea pig (Webster et al., 2013), Code SYC 391, 1:2000], followed, after extensive washing by incubation in Alexa Fluor 488 goat-anti guinea pig IgG (Invitrogen) 1:500. Preparations were mounted on cavity slides using Vectashield (Vector Labs. United Kingdom), coverslipped, sealed with nail varnish, and images collected and Z-stacked at 5 μm intervals on a Zeiss 710 confocal microscope equipped with Zen Black edition software (Carl Zeiss, Jena, Germany) or a Leica TCS SP5 confocal microscope (Leica Microsystems, Wetzlar, Germany).

Whole mount *in situ* hybridizations were performed using digoxigenin labeled riborobes as previously described (Wilcockson et al., 2011). Probe synthesis was performed using primers detailed in Supplementary Table S1. Preparations were mounted on cavity slides in 50% glycerol/PBS, sealed as described and stacks of several (usually 4–6) planes of focus imaged using Helicon Focus 6 (HeliconSoft, Karkiv, Ukraine). Images were cropped, resized and adjusted for brightness and contrast using Adobe Photoshop CC2017 and CorelDraw 2014.

### DH31 Time-Resolved Fluoroimmunoassay

Milligram quantities of affinity purified anti DH31 IgG were further purified on a Sepharose CL-4B protein A column as previously described (Morris et al., 2010), concentrated on Vivaspin 6 ultrafiltration cartridges (Sartorius Stedim Biotech, Germany) to *ca*. 10 mg/ml and stored in small aliquots (100 μg) at -20°C. Further milligram quantities of similar preparations were biotinylated and purified as previously described (Morris et al., 2010) diluted to 5mg ml^-1^ and stored at -20°C. High protein binding microplates (Costar 3590, Corning, VWR International, United Kingdom) were coated with 100 μl/well 10 μg ml^-1^ of the affinity purified IgG in 0.1 M bicarbonate buffer, pH 9.3, overnight at 4°C, washed 3x in the same buffer, and blocked for 1 h (0.1% BSA in 50 mmol l^-1^ Tris pH 8.0 containing 0.85% NaCl). Standard DH31 dilution series were 125–0.125 fmol/100 μl, dissolved in assay buffer (50 mM sodium phosphate pH 7.5, 0.15 M NaCl, 10 mM MgCl_2_, 0.05% casein). Standards and samples (100 μl) were incubated at 4°C overnight, followed by washing (5x, Delfia wash buffer, Perkin Elmer, Waltham, MA, United States), and incubated in 100 μl/well biotinylated antiDH31 IgG, 2.5 μg ml^-1^ for 6 h, RT. Plates were then washed (5x), and incubated (2h, RT) with 100 μl per well Europium-labeled streptavidin (Perkin Elmer), diluted to 100 ng ml^-1^ in proprietary assay buffer (Perkin Elmer). After washing (5x), enhancement solution [50 μl per well (Perkin Elmer)] was added, followed by orbital shaking (2 min). Time-resolved fluorescence of Europium was then measured on a Mithras LB940 microplate reader (Berthold Technologies GmbH, Bad Wildbad, Germany) and data reduction and analysis was performed using MikroWin v5.18 (Mikrotek Laborsysteme GmbH, Overrath, Germany).

To determine antiserum specificity, batches of 10 sub-oesophageal ganglia (*sog*), (this part of the nervous system contained large quantities of DH31 as visualized by IHC) were homogenized by sonication in ice-cold 2 M acetic acid, the supernatant dried by vacuum centrifugation and reconstituted in PBS. This extract was purified on 500 mg Strata X polymeric reverse phase cartridges (Phenomenex, Macclesfield, United Kingdom), using 40% isopropanol as eluant as described previously (Webster et al., 2013) and dried. This extract was then reconstituted in 2 M acetic acid and separated by high performance liquid chromatography (HPLC; Dionex Summit, Dionex, CA). Conditions were: 4.6 mm × 300 mm Jupiter C_18_ 300 Å column (Phenomenex), 20–60% solvent B over 40 min. 1 ml/min, detection at 210 nm, solvent A, 0.11% TFA; solvent B, 60% acetonitrile, 0.1% TFA. Fractions (1 ml) were dried, reconstituted in assay buffer to give (nominally) 1 *sog* eq. 100 μl^-1^. Injections of 250fmol DH31 served as standards.

### Bioassay

The cardioactivity of C. *maenas* DH31 was determined on semi-isolated heart preparations. Crabs (*ca.* 65mm carapace width) were ice-anaesthetized and rapidly decerebrated before removing all limbs and the dorsal carapace to expose the heart and pericardial cavity. The heart was connected to a force transducer (MLT0210/A) via a micro fishing hook (size 28) and fine nylon (0.08 mm) monofilament. Connection to a PC with Chart 4.0 was via a Bridge Pod (ML301) and Powerlab 4/20 (AD Instruments Pty. Ltd., Castle Hill, NSW, Australia). Transducer gain was set at maximum sensitivity (200 μV). Heart preparations were initially perfused with *C*. *maenas* physiological saline (Saver et al., 1999) at room temperature (20°C). Once a stable output was achieved, hearts were firstly perfused with 1ml saline, followed by increasing concentrations of DH31 (10^-9^–10^-6^ M), recording for approximately 2 min at each concentration.

## Results

### DH31R and DH31 Analyses

**FIGURE 1:**
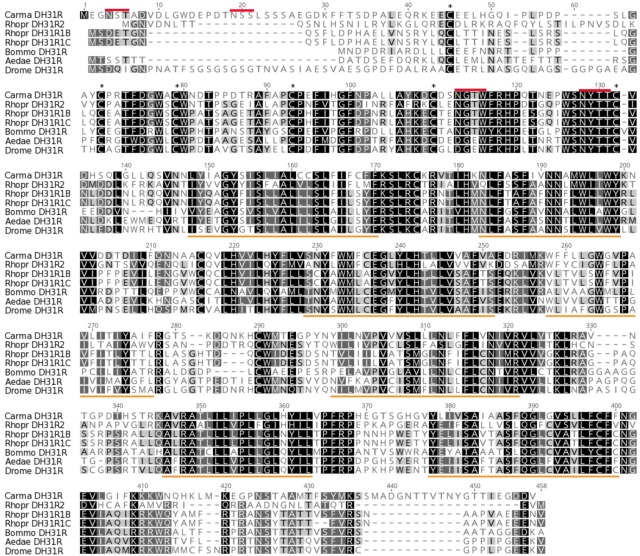
DH31R amino acid alignments of *C. maenas* DH31R (Acc No: MH331892) compared with other functionally deorphanized insect homologs: *Rhodnius prolixus* DH31R2B, DH31R1B and DH31R1C KF446640, KC660149, KC660150; (Zandawala et al., 2013); *Bombyx mori* DH31R, NP_001127732 (Iga and Kataoka, 2015); *Aedes aegypti* DH31R, JQ045343; (Kwon and Pietrantonio, 2013); and *Drosophila melanogaster* DH31R NM_165979 (Johnson et al., 2005). For ease of interpretation, overall comparative amino acid identities are indicated by black (100%), dark gray (80–99%), light gray (60–79%) and white (less than 60%). The 6 conserved cysteine residues in the N-terminal extracellular domain are marked by asterisks, the seven predicted transmembrane regions for all DH31Rs are underlined in orange. Putative *N*-glycosylation sites on the extracellular N–terminal domain of *C. maenas* DH31R are indicated by red lines.

**FIGURE 2:**
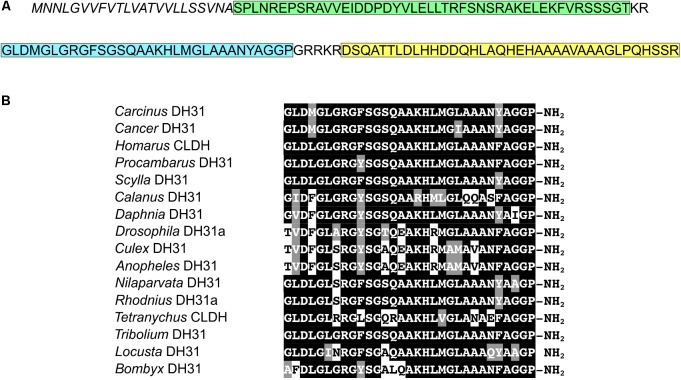
DH31 sequences. **(A)** Sequence of *C. maenas* DH31 precursor peptide. Signal peptide, italics; precursor-related peptide, green; DH31, blue; precursor-associated peptide, yellow. **(B)** Amino acid sequence alignments of diuretic hormone-31 (DH31) (with original appellations; CLDH = calcitonin-like diuretic hormone) from some representative arthropod species. Identical residues are shaded black and conserved substitutions gray. (GenBank accession numbers, contig id. and/or source information is indicated for each species. *Carcinus maenas* (MH331891); *Cancer borealis*, GEFB01004916 (Christie and Pascual, 2016); *Homarus americanus*, ACX46386.1, (Christie et al., 2010); *Procambarus clarkii* GBEV01003269.1 (Christie and Chi, 2015); *Scylla paramamosain* ALQ28573.1, (Suwansa-ard et al., 2015); *Calanus finmarchicus* comp121972_c0_seq1 (Christie et al., 2013); *Daphnia pulex*, EFX90445 (Colbourne et al., 2011); *Drosophila melanogaster*, NP_523514.1 (Smith et al., 2007); *Culex tarsalis*, JAV34908.1; *Anopheles gambiae*, XP_321755.3 (Holt et al., 2002); *Nilaparvata lugens*, BAO00939.1 (Tanaka et al., 2014); *Rhodnius prolixus*, AEA51302.1 (Zandawala et al., 2011); *Tetranychus urticae* (XP_015781297); *Tribolium castaneum*, EEZ99367.2 (Richards et al., 2008); *Locusta migratoria*, AKN21237 (Hou et al., 2015); *Bombyx mori*, NP_001124379.1 (Ou et al., 2014).

Complete cDNA sequences encoding GPCRs were identified from tBLASTn searches of our *Carcinus* transcriptomes. The individual SRA codes are: SRX 3280798-805, SRX 3280810-814, SRX 3280830-846 and are deposited in the NCBI SRA archive as BioProject PRJNA400568. The transcriptome shotgun assembly project has been deposited at DDB/EMBL/GenBank under the accessions: GFX00000000 (*Carma*_CNS-transcriptome). One was functionally identified as the DH31 receptor (Acc. No: MH331892). Full-length cDNA sequence encoding a DH31 was identified (Acc. No: MH331891), which was identical to that previously isolated using a conventional cloning strategy with degenerate primers, 5′ and 3′RACE (Webster, unpublished). Deduced amino acid sequences for DH31R and comparisons of functionally identified DH31 receptors from insects are shown on **Figure 1** and the prepro- DH31, with comparisons with selected crustacean and insect mature peptides are shown on **Figure 2**, together with a cladogram for crustacean DH31s.

The DH31R (ORF 1314 bp, 438 amino acids) had typical characteristics of secretin-like (Class B1) GPCRs (Prosite): Seven transmembrane domains were predicted (TMHMM 2.0 server) and likewise pfam analysis predicted a seven transmembrane interval, and six conserved cysteine residues in the n-terminal extracellular domain. Four potential *N*-glycosylation sites were predicted for the N-terminal extracellular domain (NetNGlyc 1.0 server). Multiple sequence alignments with other cloned (and functionally identified) insect DH31Rs show, as expected, high levels of conservation.

The sequence of a single DH31 like prepropeptide was identified in our neurotranscriptomes. This was encoded by a 300bp ORF, encoding a signal peptide (23 residues), a 47 residue amidated precursor-related peptide, the 31-residue mature DH31 with amidation and tetrabasic cleavage sites in common to all DH’s, and a 23 residue precursor-associated peptide (**Figure 2A**). Representative examples of crustacean and insect DH31s showed very high sequence similarity (**Figure 2B**).

### Functional Confirmation and Expression of DH31R

**FIGURE 3:**
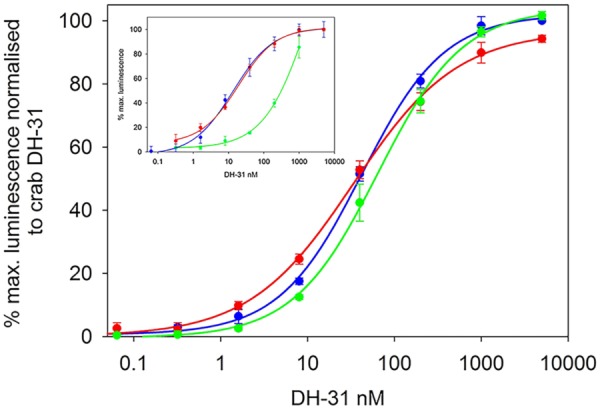
Functional confirmation of *C. maenas* DH31R. Dose-response curves of the luminescence response following addition of various DH31s to CHO-K1-Aeq-Gα16 cells transiently expressing DH-31R. *C. maenas* (blue), *Tribolium castaneum* (green), *Diploptera punctata* (red). EC_50_ *ca*. 30nM. Values are means of *n* = 4 ± 1 SD. Inset: dose-response curves of the luminescence response following addition of *C. maenas* DH31 to simultaneously transfected CHO-K1 Aeq-Gα16 (blue) or Gq (red), DH31R transfected cells. EC_50_ *ca*. 15 nM. Dose-response curve for Alexa-488 *Drosophila melanogaster* DH31 applied to DH31 transfected CHO-K1-Aeq-Gα16 cells (green). EC_50_ *ca*. 350 nM. Values are means of *N* = 4 ± 1 SD.

Transient expression of cloned DH31R into CHO-K1-Aeq cells expressing the Gα-16 subunit showed similar luminescence responses to those where the receptor was transfected into cells expressing the G*q* protein with EC_50_ values of around 15 nM. (**Figure 3**). When Gα-16 cells were transfected with DH31R and exposed to insect DHs (*Tribolium castaneum, Diploptera punctata*), strikingly similar dose response characteristics to that from *C. maenas* DH31 were observed (**Figure 3**). In these (simultaneously performed) experiments EC_50_ values were quite similar to those (from another set of independently performed transfections) shown in **Figure 3** at around 30 nM. No luminescence responses were observed, even at micromolar levels with *R. prolixus* CRF-like DH or *C. maenas* PDH-1 or with empty vector controls. A reasonable luminescence response was obtained with Alexa 488-labeled *Drosophila* DH31, but with an EC_50_ of 350 nM.

**FIGURE 4:**
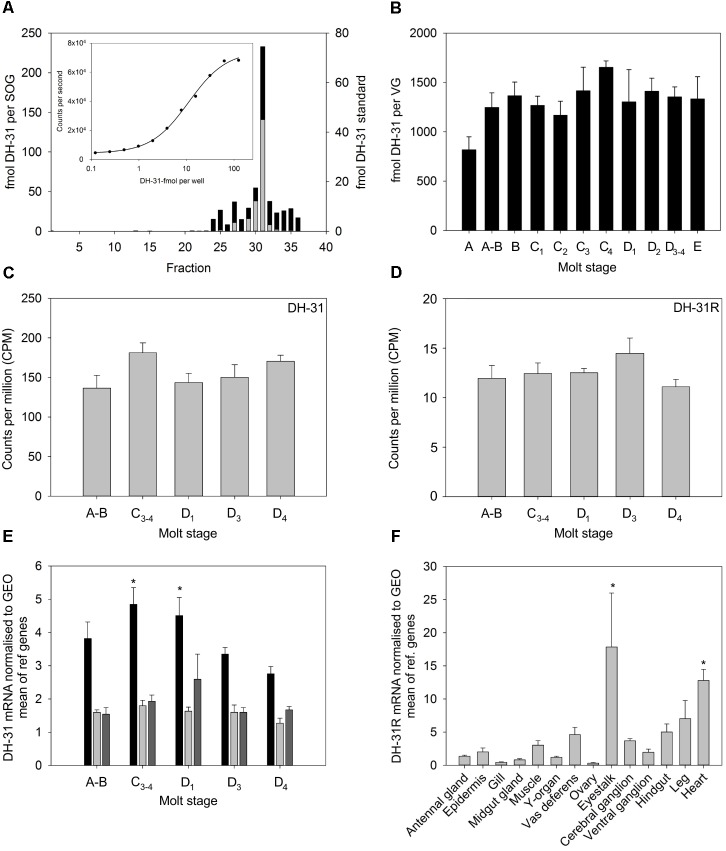
Tissue distribution and abundance of DH31 and DH31R during the molt cycle of *C. maenas*. **(A)** HPLC- TR-FIA identification of DH31 from 10 sub-oesophageal ganglia (SOG) extracts. Sample preparation, cleanup, chromatographic conditions and TR-FIA as detailed in the text. Black bars, SOG extract, gray bars, DH-31 peptide standard (250 fmol). **(B)** Quantification of DH31 in ventral ganglia extracts during the molt cycle, *n* = A, 4; A-B, 5; B-C_3_, 6; C_4_, 4, D_1_ 3; D_2_, 10; D_3-4_ 8; E, 3. **(C,D)** *In silico* quantification of DH31 and DH31R from whole CNS mRNA *n* = 5 at each molt stage. **(E)** qRT-PCR quantification of DH31 expression in eyestalk (black), brain (light gray), and ventral ganglia (dark gray) during the molt cycle. Values are means (*n* = 5) ± 1SEM, normalized to the geometric mean (GEO) of elongation factor-1 and ubiquitin ligase mRNA. Asterisks indicate significantly higher (*p* < 0.05, ANOVA, Holm–Sidak pairwise comparison) levels of DH31 in the eyestalk at molt stages C_3-4_ and D_1_ compared to D_4_. **(F)** qRT-PCR showing tissue distribution and abundance of mRNA encoding DH31. Values are means of *n* = 5 ± 1SEM. Normalizers were GEO means as described above. Asterisks indicate significantly higher levels of expression in the heart and eye tissues, compared to those in the ovary, gill, midgut gland, (*p* < 0.05, Kruskal–Wallis ANOVA, Tukey pairwise comparisons).

Quantitative PCR of DH31R from RNAs extracted from a variety of tissues showed detectable but quite low expression in many tissues. For nervous system tissues, the eyestalk expressed the highest (but quite variable) levels of receptor transcripts, and it was notable that muscle tissues (hindgut, leg, heart), moderate levels of receptor were expressed (**Figure 4F**). *In silico* quantification of DH31R expression in nervous tissue (eyestalk, cerebral and ventral ganglia) once again reflected a rather low level of expression (10–15 counts per million reads), and this was invariant throughout the molt cycle (**Figure 4D**).

### Measurement of Molt Stage and Tissue Specific Expression of DH31

Development of a TR-FIA for DH31 allowed measurement of the peptide in CNS tissues. The assay had a detection limit of around 1 fmol per well. HPLC of nervous system extracts, followed by TR-FIA showed a peak corresponding with authentic peptide, but this was broader than expected, which may have been the result of Met oxidation (M_4,20_), peptide cleavage and/or non-specific binding (**Figure 4A**). Measurement of DH31 levels in ventral ganglia showed little variation during the molt cycle (**Figure 4B**), and transcript level was similarly invariant, whether measured *in silico* (**Figure 4C**) or by qPCR, except a significant (*p* < 0.05) increase transcript levels in eyestalk neural tissues in intermolt (C_3-4_) and early premolt (D_1_), when compared with those in late premolt (D_4_), (**Figure 4E**).

### Biological Activity of DH31

**FIGURE 5:**
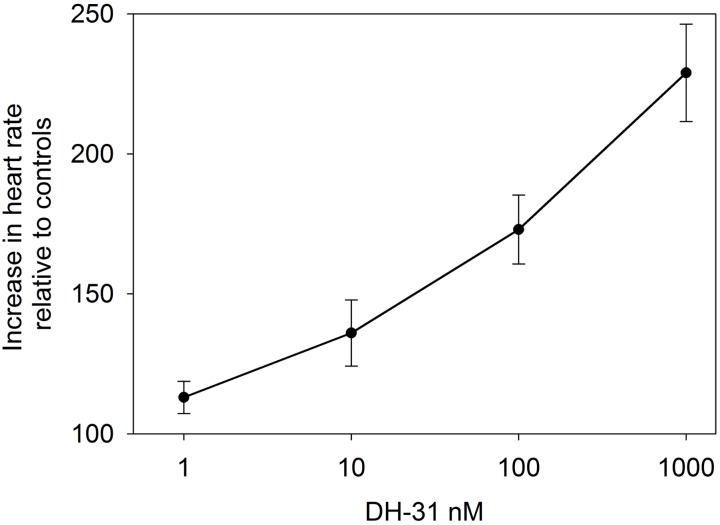
Effect of DH31 on semi-isolated heart preparations of *C. maenas*. Values are means of *n* = 5 ± 1 SEM.

Superfusive application of DH31 to semi-isolated heart preparations demonstrated a clear dose-dependent cardioacceleratory (chronotropic, but not inotropic) response. Micromolar application of DH31 led to more than a doubling of heart rate compared to controls, and the minimum dose resulting in an increase in heart rate was between 10 and 100 nM (**Figure 5**).

### Immunohistochemistry and *in Situ* Hybridization of DH31 Peptides and Transcripts in the CNS

**FIGURE 6:**
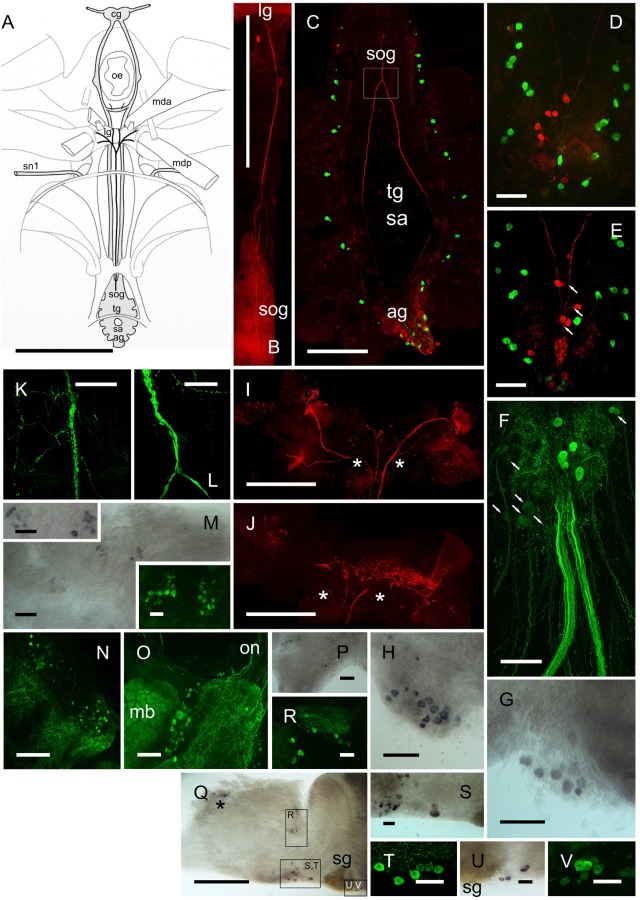
DH31 in the central nervous system. Detection of peptide by ICC and confocal microscopy **(B–F,N,O,R,T,V)** or mRNA by ISH **(G,H,M,P,Q,S,U)**. **(A)** Orientation cartoon showing position of CNS with reference to internal muscular and endoskeletal anatomy relevant to that of DH31 neural structures. **(B)** Composite image showing median DH31 nerve exiting the sog and terminating anteriorly on the “ligament” lg. **(C)** Composite image highlighting gross neuroanatomy of the ventral ganglionic mass, doubly labeled for BURS (green) and DH31 (red). Note prominent fascicles of axons ascending through the tg, and combining to form a single tract in the sog (boxed). **(D,E)** BURS (green) and DH31 immunoreactive neurons in the abdominal ganglion. 5–6 heavily labeled unpaired dorsal perikarya were usually observed, which projected fine axons to the axon tract (arrows, **E**). **(F)** Composite image showing five heavily labeled DH31 perikarya, and a number of ventral, lightly labeled cell bodies. In all preparations, labeling of the numerous ventral cells was always light and variable. Note two large, intensely labeled abdominal nerves. These could be traced to the hindgut (**Figure 8B**). **(G,H)** *In situ* hybridization of ag whole mounts. Note variability in labeling (cell number and intensity) in two preparations. **(I,J)** Composite views of DH IR structures terminating on the “ligament”. **(I)** Shows a composite of dorsal planes of focus, **(J)** deeper, ventral focus planes. Asterisks indicate continuations of axonal projections. **(K,L)** Detail of axon tract junction of sog indicated in box, **(C)**. Note numerous branched and beaded fibers on the surface of the sog. **(M)** DH31 neurons, ventral midline of cerebral ganglion, visualized by ISH and ICC. **(N)** Numerous small DH31 immunopositive cell bodies and branching fibers on the anterio-dorsal margin of the protocerebrum. **(O)** A group of cell bodies between the mushroom body (mb) and central protocerebrum. Note variation in labeling intensity, and intense labeling of the mb. Several fibers project from the on to the protocerebrum. **(P)** ISH of tritocerebrum showing cell bodies on the ventral margin of this structure. **(Q)** Overview of eyestalk ISH. Boxes show neuronal groups identified, either shown as enlargements **(S,U)** or by IHC of other preparations **(R,T,V)**. Asterisk in **(P)** indicates non-specific labeling adjacent to X-organ. ag, abdominal ganglion; cg, cerebral ganglion; lg, ligament; mb, mushroom body; mda, *musculus dorsoventralis anterior*; mdp, *musculus dorsoventralis posterior*; oe, esophagus; on, optic nerve; sa, sternal artery (foramen); sog, sub-oesophageal ganglion; sg, sinus gland; sn1, segmental nerve 1. Scale bars **(A,B)** 10 mm; **(B)** 1 mm; **(I,J,P)** 500 μm; **(D,E)** 250 μm; **(F–H,K,L)** 200 μm; **(M–O,Q)** 100 μm; **(M)** inserts, **(R–V)**, 50 μm.

Immunolabeling of the CNS ganglia (eyestalk, cerebral ganglia, fused thoracic ganglion) with affinity purified anti-DH31 antiserum raised against native *C. maenas* DH31 revealed an extensive and novel neurosecretory system, in particular that of the abdominal ganglion (**Figures 6B–F**). On the dorsal surface of the abdominal ganglion (*ag*), a variable number (usually 5–6) large unpaired median neurons (50 μm), which are distinct from the more numerous BURS/CCAP neurones, project ascending axons to form prominent, intensely labeled dorsally projecting tracts (**Figure 6C**) surrounding the sternal artery foramen. The tracts then fuse to form a single fascicle of axons in the sub-oesophageal ganglion (*sog*) and a complex system of fine branching axons, covering the dorsal aspect is visible (**Figures 6K,L**). This tract then leaves the CNS projecting anteriorly, forming a single fine median nerve (ca. 100 μm diameter) which branches extensively over an endoskeletal “ligament” posterior to the esophagus onto which several small muscles attach (see the cartoon **Figure 6A** for orientation and overview), - the *mda* and *posterior* muscles (*mda, mdp* nomenclature according to Maynard, 1961). On the surface of the “ligament”, there are extensive branches of this median nerve, and fine branching arborisations, which form a large (ca. 1.5 mm) dendritic field (**Figures 6I,J**). Due to the difficulty of dissection, and intimate association of the fine branching axon projections with the endoskeleton, more detailed neuroanatomical investigations were not started at this time, but the gross anatomy is suggestive of a neurohemal area.

Ventrally, numerous, rather weakly immunopositive neurons were observed in the *ag*. Some of these appeared to project fine axons to the abdominal nerves, however, the number and association of these neurones, in particular was difficult to ascertain, since a complex field of branching IR fibers pervade much of this ganglion (**Figure 6F**). A number of ventral perikarya were always observed, but since they were invariably rather weakly immunolabeled, their number was difficult to determine. Likewise, *in situ* hybridization studies showed considerable variation in labeling intensity (**Figures 6G,H**), which was always moderate, and it would seem that this variation, whether determined by ISH or IHC is a feature of the DH31 neurons. The association of many fine axons with the two central abdominal nerves was noteworthy (**Figure 6F**) since these nerves project to the proximal hindgut (**Figure 8B**). Immunohistochemistry of whole-mounted eyestalks and cerebral ganglia revealed a number of small, mainly weakly immunopositive (inter)neurons (**Figures 6M–V**), but background was an issue in these tissues, particularly for the cerebral ganglion where labeling of structures such as the mushroom bodies (**Figure 6O**) made stacked maximum intensity projection confocal images difficult to interpret. Again, ISH was performed to confirm identity of these, and in many instances correspondence between cell groups could be established, although due to variation in hybridization and immunolabeling intensity, it was, once again, difficult to confirm individual neuron identities, but these could be tentatively established (with regard to location) in many instances (**Figures 6M,R–V**). ISH using sense probes (results not shown) did not reveal any hybridization signals.

**FIGURE 7:**
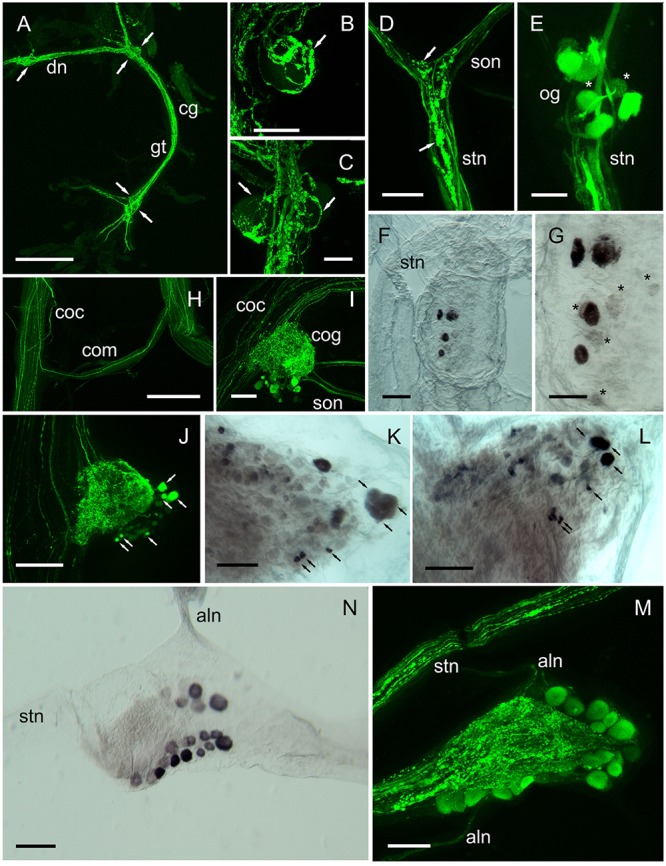
DH31 in the peripheral nervous system. Detection of peptide by ICC and confocal microscopy **(A–E,H–J,M)**, or mRNA by ISH **(F,G,K,L,N)**. **(A)** Cardiac ganglion showing extensive, strongly immunopositive axonal projections. Arrows show positions of 4 (of 5) prominent neuronal somata, which were not labeled. **(B,C)** Detail of neuronal cell bodies in the anterior **(B)** and posterior ganglionic tract **(C)**, showing extensive dendrites and secretory boutons closely associated with the surface of these cells (arrows). **(D)** Immunopositive fibers and secretory boutons (arrows) at the junction between the superior oesophageal nerve and the stomatogastric nerve. **(E)** Oesophageal ganglion showing 5–6 immunopositive and two weakly labeled somata (asterisks). **(F,G)** ISH of the oesphageal ganglion. Note 4 strongly hybridizing, and several weakly hybridizing somata (asterisks). **(H)** Circum-oesophageal connectives and commissure. Note a single strongly immunopositive axonal projection, which ascends via the opposite connective. **(I,J)** Circum-oesophageal connective ganglia. Note large and small immunopositive somata (arrows), many of which can be identified by ISH although double labeling by ISH and IHC was not performed, the arrows indicate potential colocalization of transcript and peptide **(K,L)**. Note immunopositive axon profiles in the superior and inferior oesophageal nerves, and branching dendrites throughout the commissural ganglia. **(K,L)** ISH of connective ganglia. Black arrows indicate neuronal somata that can be identified by ICC. **(M)** Stomatogastric ganglion. Note prominent beaded axon profiles in the stomatogastric nerve, and dendrites in the central region of the ganglion. *ca.* 20 immunopositive somata can be identified in this preparation. **(N)** ISH of the stomatogastric ganglion. In this preparation *ca.* 19 hybridizing somata are visible. aln, anterior lateral nerve; cg, cardiac ganglion; cog, circum-oesophageal ganglion; coc, circum-oesophageal connective; com, commissure; dn, dorsal nerve; gt, ganglion tract; ion, inferior oesophageal nerve; son, superior oesophageal nerve; stg, stomatogastric ganglion. Scale bars: **(A,H)** 500 μm; **(I,J)** 200 μm; **(D,F,K–N)** 100 μm; **(B,C,E,G)** 50 μm.

Expression of DH31 peptide and transcripts were also investigated in peripheral CNS tissues, as summarized on **Figure 7**. DH31 immunoreactive (IR) structures were observed in the cardiac (*cg*), commissural (*cog*), and stomatogastric ganglia (*stg*) and IR neuronal terminations were observed in muscular systems associated with the fore- and hindgut. With regard to the cardiac ganglion (*cg*), very prominent beaded fiber axon profiles and terminal varicosities were abundant, throughout this tissue (**Figures 7A–C**). It was noteworthy that whilst the five large cells identified as motoneurons (*ca.* 50 μm) were easily identifiable in the dorsal nerves (2) anterior ganglion tract (1, occasionally 2) and posterior ganglion tract (2), these somata were immunonegative. However, complex, beaded immunopositive varicosities appeared to cover these somata (**Figures 7B,C**). The four small pacemaker cells, that are located at the posterior of the *cg* were not visible (they are deeply embedded in neuropil) and were immunonegative in our preparations. Neuronal input to the cardiac ganglion was occasionally observed, via a tiny dorsal nerve in the *Po*, but this could not be traced either proximally or distally. Furthermore, immunopositive axons could not be observed in the segmental nerves.

**FIGURE 8:**
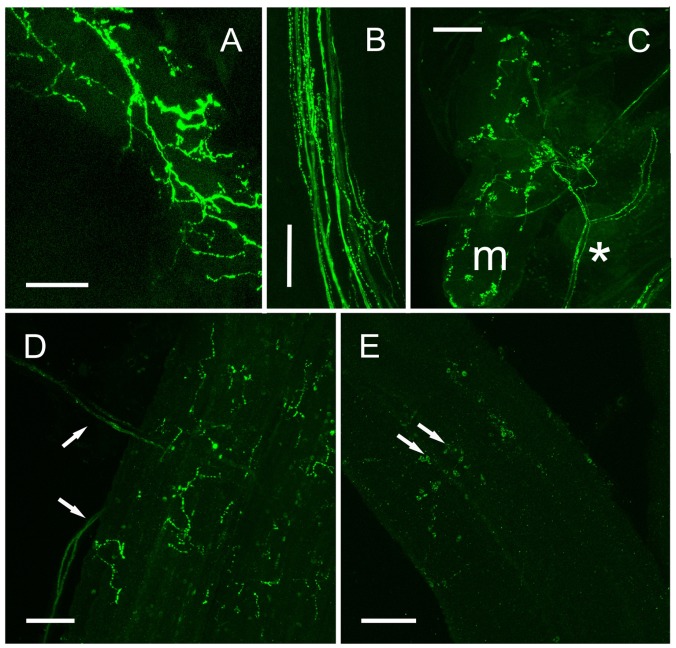
DH 31 in fore- and hindgut musculature. **(A)** Posterior hindgut muscles showing extensive innervation via branching axons and secretory boutons. **(B)** Large lateral abdominal nerve containing many immunopsitive axons and beaded fibers which innervate the posterior hindgut. **(C)** Small oesophageal muscle (m) showing many secretory boutons innervated via a branch of a small nerve (asterisk), which could be traced to the oesophageal ganglion. **(D)** Cardiopyloric valve 2b muscle, showing axons innervating this muscle (arrows), and fine branching immunopositive fibers and boutons. **(E)** Skeletal (limb) muscle does not show DH31 immunoreactive nerve fibers. Confocal set at maximum gain. Arrows show non-specific immunoreactivity of adherent hemocytes. Scale bars 100 μm.

Immunopositive axon profiles were prominent in the stomatogastric nervous system. In particular, the superior oesophageal nerve (*son*) and *stn* (**Figure 7D**), where prominent terminal boutons, suggestive of release sites were abundant at the junction between the *son* and *stn*. In several preparations, the small *og* was dissected. Immunolabeling revealed 6–8 immunpositive perikarya, which appeared to project axons along the *stn* to the *stg*, but often some showed weak, rather granular labeling (**Figure 7E**). Nevertheless, ISH of the *og*, revealed strong hybridization signals in four cells, and much weaker signals in 4–5 further cells (**Figures 7F,G**). Many descending axons were visible in the circum-oesophageal connectives (*coc*), and some fibers projected contralaterally, and ascended in the opposite tract (**Figure 7H**). However, these axon profiles were too fine to permit further analysis. Nevertheless, a prominent feature of the *coc* was the intense labeling of axonal branches and perikarya in the *cog*, where a number of large and small perikarya were always seen (**Figures 7I,J**). Comparison of ISH with IHC of the *cog* showed that the IR profiles could be matched to the hybridization signals (**Figures 7K,L**) in the majority of instances, but once again, the variation in expression of peptide or mRNA made further interpretation rather difficult. *stg* preparations showed intense immunolabeling of axon profiles in the *stn* that exhibited a complex branching anatomy in the neuropil of the *stg*. Around 20 neuronal cell bodies appeared to be strongly, but variably immunopositive (**Figure 7M**). To preclude this labeling pattern as an artifact, which could be due (for example) to leaching of peptide from the branching axon profiles of the *stn* during dissection/fixation, we performed ISH on the *stg*. Our preparations invariably showed 19–20 hybridizing somata, with (again) considerable variation in signal strength (**Figure 7N**). IR axonal processes in the dorsal ventricular nerve (*dvn*) were very faintly labeled, but labeled axonal profiles were seen in the anterior lateral nerve (*aln*) (**Figure 7M**) IHC of muscle preparations of gut tissues showed intense labeling of fine branching dendrites: hindgut muscles were innervated by two lateral nerve bundles containing a number of axon profiles (often beaded) which terminated on the hindgut longitudinal muscles (**Figures 8A,B**). When dissecting stomatogastric nervous system preparations, small muscle fragments often remained on these; they were possibly the lateral anterior and interior ventricle dilators (*c4*, *cv1*) (nomenclature: Maynard and Dando, 1974). On the surface of these muscles intense labeling of terminal boutons were observed, together with axon profiles within a small nerve (**Figure 8C**). Preliminary investigations also revealed DH31 innervation on the cardio pyloric valve 2b muscle (*cpv2b*) (**Figure 8D**) and these seemed to be specific- similar profiles were never observed on limb muscle preparations (**Figure 8E**).

## Discussion

In this study we have isolated and functionally confirmed the cDNA sequence encoding the DH31 receptor in the crab *C. maenas* for the first time, and determined its tissue- and stage-specific expression patterns. Furthermore, we have identified its cognate ligand in terms of both tissue distribution, neuroanatomy, and biological activity, our overarching objective being to understand the function of this newly discovered neuroendocrine system in crustaceans.

Our *C. maenas* neurotranscriptomes revealed just one DH31R transcript which is a member of the secretin (Class B1) GPCR family; as are other identified arthropod DH31 GPCRs which show not only high sequence identity in the transmembrane regions, but also in the extracellular N-terminal region where the six conserved Cys residues are located, which are typical for the secretin receptor family. Additionally, characteristic potential *N*-glycosylation sites occur in this region-four were predicted for *C. maenas*, and it is noteworthy that the last two (NGTW, NYTT) are common to the majority of DH31Rs. In comparison with other deorphanized insect DH31Rs, for the kissing bug, *Rhodnius prolixus*, several DH31R splice variants exist; these arise from two genes orthologous to the DH receptor of *Drosophila* (CG17415) (Johnson et al., 2005) and an orphan (CG4395) previously annotated as *hector* (Zandawala et al., 2013). In the heterologous assay system used for *R. prolixus* receptor identification the R1B isoform was activated by much higher concentrations of DH31 [EC_50_ 200–300 nM, than the R1B isoform (15 nM)]. It seems possible that there may have been gene duplication of this receptor in insects, yet with regard to identified but as yet functionally uncharacterized crustacean DH31 receptors, only single transcripts have so far been identified (*D. pulex, C. finmarchicus*) (Colbourne et al., 2011; Christie et al., 2013), so it is probably premature to speculate on this event in an evolutionary context. For the *Drosophila* DH31R it has been previously established that function of this receptor is greatly enhanced by co-expression of mammalian or *Drosophila* RCP or mammalian receptor activity- modifying proteins (RAMPs) Johnson et al. (2005). Indeed, it is well established that mammalian CLRs interact with RAMPs, as pharmacological switches, chaperones and in receptor trafficking (review: Hay and Pioszak, 2016), and might be a vital and universal component of Class B1/CLR/DH31 receptor signaling. Although RAMPs have yet to be identified in crustaceans, (and are absent from the *R. prolixus* genome, Zandawala et al., 2013) it seems possible that the rather modest sensitivity to DH31, might reflect the absence of these accessory proteins (although CHO cells do express low levels of all three identified mammalian RAMPs, Wootten et al., 2013). For insects, DH31s mediate their effects via increases in intracellular cAMP in dipterans (Coast et al., 2001, 2005; Furuya et al., 2000) and *R. prolixus* (Te Brugge et al., 2008), and in the latter it has been proposed that Gs signaling leads to cAMP production and binding to a cyclic nucleotide gated channel, resulting in Ca^2+^ influx (Zandawala et al., 2013). Whilst receptor activation in the CHO cell bioassay used here signaled via G*q* resulting in Ca^2+^ release, further details of homologous signaling pathways now need to be established by measurement of direct responses of target tissues with respect to calcium (many class B1receptors signal via this messenger (Review: Mayo et al., 2003) and cAMP levels after *in vivo* or *in vitro* administration of DH31 in *C. maenas*.

Given the sequence conservation of DH31 across the phylum Arthropoda, it was unsurprising that all three DH31s tested in the CHO cell bioassay gave essentially identical responses, and it was noteworthy that the N-terminally modified *Drosophila* DH31 that had been fluorescently labeled (Alexa 488) also gave a reasonable response in this bioassay. Thus, since this N-terminal fluorescent analog does not abrogate receptor activation, the elegant techniques using such analogs to report receptor localization (in insect MT), described by Halberg et al. (2015) might well be profitably used to determine DH31R expressing cells in crustacean tissues. At this juncture it is worth mentioning that a large part of the N-terminus of DH31 does seem to be essential for receptor binding and biological activity, since a truncated analog of *R. prolixus* DH 31 (17-31) is inactive in the CHO cell luminescence and hindgut contraction assay (Zandawala et al., 2015).

In *Drosophila*, the PDFR is also activated (in heterologous assays) by DH31 (Mertens et al., 2005). And it is now becoming apparent that PDF and DH31 signaling are implicated in a variety of clock-related behaviors (Kunst et al., 2014; Goda et al., 2016, 2018). We found that DH31 has no activity when applied to CHO cells expressing either of the two candidate *C. maenas* PDHRs (our unpublished results and PDH-1 does not activate the DH31 receptor. Thus, although may be premature to speculate, it seems possible that the overlap of peptide/receptor activities seen in insects, might not occur in crustaceans and may be related to the rather more circumscribed roles of PDHs in crustaceans, where their primary functions appear to be in control of pigment migration in the epidermis and retina (although pigment migration is clearly under circadian clock control in the isopod *Eurydice pulchra*, Wilcockson et al., 2011).

Analysis of the spatial expression of DH31R revealed that it was widely transcribed in most of the tissues examined, in particular, the eyestalk and heart and leg muscles. Whilst the expression in the latter might be associated with the role of DH31 as a myoactive peptide, as is also suggested by its cardioacceleratory activity, it is likely that DH31 fulfill quite a number of roles in crustacean physiology. Although DH31 peptide expression seemed to be invariant during the molt cycle, in the nervous tissues examined, with the exception of some stage specific expression of transcript in eyestalk tissues (stage specific peptide levels in eyestalk or brain tissues were not examined), the involvement of DH31, if any, in molt control might seem to be unimportant, when compared to the notable changes in expression of peptide levels in the CNS for CCAP and BURS (Webster et al., 2013). Nevertheless, in view of the anatomy of DH31 expressing neurons, particularly those in the abdominal ganglia, that direct axons to a large, seemingly neurohemal area behind the esophagus, it seems likely that DH31 is a secretable neurohormone, and given the sensitivity of the TR-FIA described here, future progress in evaluating any role of this peptide in ecdysis will be expedited by accurate measurement of circulating DH31 levels.

Neuroanatomical studies, using both whole mount immunohistochemistry and *in situ* hybridization showed an extensive and complex array of DH31 neurons in the CNS and peripheral nervous system. Whilst the variable expression of peptide and mRNA often made interpretation challenging, for the most extensive neurosecretory system- that of the abdominal ganglion, DH31 immunoreactivity was found in a group of 5–6 dorsal unpaired neurons, quite separate from those expressing the CCAP/BURS (Webster et al., 2013), which directed axonal processes to two fascicles ascending to the sub-oesophageal ganglion, which then project a single median nerve to the surface of a region of internal exoskeleton terminating in a complex array of branching fibers forming what appeared to be a very large and extensive neurohemal area. This neurosecretory system (and indeed, the presence of the median nerve) has not previously been described in any crustacean. The morphology of the DH31 IR neurons in the abdominal ganglion was, however, not without anatomical similarity to that described for *R. prolixus*, where a group of 5 dorsal unpaired median (DUM) neurons are prominent, furthermore smaller (paired) ventral cells were observed in *R. prolixus*, together with axon profiles and neurohemal sites in the abdominal and trunk nerves (Te Brugge et al., 2005). Whilst somewhat different in overall anatomy, groups of dorsal and ventral cells and projections to the trunk and abdominal nerves are also seen in another hemipteran, the large milkweed bug, *Oncopeltus fasciatus* (Te Brugge and Orchard, 2008). Although details of the axonal projections of the smaller and more numerous ventral DH31 cells were very difficult to interpret, it was notable that fine axons were visible in the abdominal nerves in our preparations as well, and likewise, there was extensive innervation of the hindgut by many fine branching fibers. Thus, the overall anatomy seems to be homologous to that seen in *R. prolixus*. Endocrine-like cells were not visible in the hindgut of our preparations, and since the midgut of crustaceans is short, we did not investigate this. Eyestalk and cerebral ganglia tissues contained many small (presumably, inter)neurons, with rather limited labeling, either by IHC or ISH. Correspondence in expression patterns for many of these was observed, but rather heavy background in these tissues precluded further analysis at this time. Nevertheless, the commissural ganglia showed intense labeling of numerous axon profiles and cells, which projected prominent fibers along both the superior and inferior oesophageal nerves toward the stomatogastric system. This situation reflects the situation in crayfish (*Orconectes limosus*), where immunoreactive axon profiles, putative release sites and varicosities (for example at the junction of the *son* and *stn*) have been found for quite a number of neuropeptides (Skiebe, 2003 and references therein). A notable finding was that both the *Og* and *Stg* expressed DH31 and transcript in defined sets of neurons: for the *og* 4 cell bodies labeled intensely, and for the *stg* 19–20 cells were labeled, and descending axonal projections from the *stn* formed a dense IR network in the center of this ganglion. The rich peptidergic inventory and anatomy of axonal projections containing neuromodulatory peptides terminating in the *stg*, and their actions in modulating stg circuit neurons and stomach rhythms has long been established, and there is an extensive literature (review: Marder, 2012). However, for the motoneurons in the *stg*, only a few cell bodies seem to be immunopositive to neuropeptides, for example 6 for orcokinin in the crayfish *Cherax destructor*, and likewise 4 somata in the *Og* (Skiebe et al., 2002). It was therefore surprising that such a large number of these contained DH31 mRNA and peptide, and apparent colocalization of both transcript and peptide (although this was not established by co-labeling) would seem to preclude artefactual phenomena. The observation that the *Og* contains DH31 expressing neurons might also have anatomical correlates in insects; the equivalent structure (the frontal ganglion) expresses DH31 in *Bombyx mori* larvae, and it has been proposed that this peptide may modulate feeding behavior (Nagata and Nagasawa, 2017). It would now be opportune to further localize DH31 expression in the *stg* and *og* neurons by using imaging mass spectrometry, (Yu et al., 2014), and also confirming that dense bodied vesicles in these (and other neurons) could be specifically labeled using immuno-gold labeling and electron microscopy. Whilst detailed dissection of the entire set of nerves innervating the muscles of the cardiac and pyloric stomach was not attempted at this time, small nerves terminating on ventral muscles (probably *c4* and *cv1*) showed intense immunolabeled axon profiles and branching fibers terminating in secretory boutons. Furthermore, preliminary IHC studies on muscles of the cardiac (*gm 1*) and pyloric stomach (*cpv*) showed similar branching IR axonal profiles, which were entirely absent from skeletal (limb) muscle preparations. A strikingly similar series of orcokinin IR axonal profiles have been observed on the *c6* muscle group (analogous to *gm1*) in crayfish (Skiebe, 2003), which might suggest that some stomach muscles are likewise under direct modulatory control by DH31.

Whilst we did not perform any bioassays to measure the effect of DH31 on hindgut motility (we found it difficult to maintain hindgut contractions in isolated or semi-isolated hindgut preparations), DH 31 was myoactive in semi-isolated heart preparations, and in view of the neuroanatomy of DH31 in the fore and hindgut, it is tempting to speculate that it may be myoactive in crustaceans as well as insects and play a role in the modulation of rhythmic behaviors in the stomach and hindgut. The suggestion that the arthropod stomatogastric nervous system may be involved in molting behavior and ecdysis (Ayali, 2009) and given our observations on DH31 innervation of stomach muscles might well implicate it in this process since the chitinous lining of the fore and hindgut must be loosened and shed during ecdysis. Many muscles control the mouthparts (which are all shed at ecdysis), and location of the putative neurohemal tissue in the thorax may be relevant: this tissue lies directly adjacent to the *mda*, and this muscle has been suggested to be homologous to the “molting muscle” in shrimp (Knowles, 1953) by Maynard (1961). In view of the stereotyped ecdysis behaviors of crustaceans (Phlippen et al., 2000), the large DH31 neurohemal release site would seem to be anatomically ideally placed to modulate such activities.

Another surprising feature of DH31 neuroanatomy concerned that of the cardiac ganglion (*cg*). In the lobster, *H. americanus* transcripts for this peptide were not only found in the brain, eyestalk, and connective ganglia- as in this study, but were highly expressed in the *cg*- in the large cells (motoneurons), but not the small cells (pacemaker cells) and this peptide is profoundly cardioacceleratory (10^-11^–10^-10^ M) (Christie et al., 2010), in contrast to the more modest dose response threshold we observed in *C. maenas*, > 10^-8^ M. Furthermore, analysis of *cg* transcriptomes have identified 10 neuropeptide transcripts, including DH31, and IHC indicated that the motoneurons of the lobster cardiac ganglion express DH31 (Gandler et al., 2017). Whilst the dissection of intact, much smaller cardiac ganglion of *C. maenas* was technically quite challenging, since it is embedded deep in the heart musculature, with no obvious landmarks or contrast (Saver et al., 1999), several preparations were successfully dissected and immunolabeled to visualize DH31 IR. These revealed strongly labeled fibers and varicosities throughout the ganglion. Input to the *cg* appeared to be via a rather fine dorsal nerve, originating from the *po*. We could not see obvious input to the *po* via the segmental nerves as would be expected, but it is possible that input to the *po* via the segmental nerves was from the branching fibers seen in the *sog.* The large, highly visible motoneurons were never immunopositive, rather, their somata were covered with a network of blebbed IR fibers and boutons, suggestive of release sites. It is interesting to note that in the classic, detailed morphological study on innervation of the heart in decapod crustaceans, using methylene blue preparations, Alexandrowicz (1932) mentions that, in crabs, the large cells (motoneurons) in the cardiac ganglion are surrounded by “numerous beaded fibers… surrounding the cells in a kind of basketwork,” and that the nerves innervating the cardiac ganglion show extensive branchings and beaded varicosities along the *cg* trunk. Thus, the neuroanatomy shown here is strikingly similar, and suggest that the neural inputs described over 80 years ago may be those containing DH31. In this context, it is interesting to note that the cardiac ganglia of *H. americanus* show characteristic axon profiles and synapses rich in dense-cored vesicles in the pacemaker cell region of the ganglion (Morganelli and Sherman, 1987). Thus, it seems likely that peptides are released from intrinsic synapses in the cardiac ganglion, and it would be interesting to confirm this for DH31 at the immuno-electron microscopic level. Furthermore, given that isolated *cg* or cultured motoneuron somata can be readily studied electrophysiologically, and bursting patterns analyzed after topical addition of a number of neuropeptides known to be cardioactive *in vivo* (Saver et al., 1999), such experiments would be interesting to do using DH-31 to define its role in cardiac central pattern generation and cardioactivity.

This is the first study to identify, functionally confirm and determine tissue expression of a GPCR for DH31 in a crustacean, and to show that this peptide has an extensive distribution in the CNS. We have identified novel neural structures, neurosecretory and putative neurohemal release sites. By considering both the neuroanatomy and myoactivity of DH31 and distribution of its receptor, we propose that this peptide signaling system, which controls a wide variety of processes in insects, might have fundamental roles to play in co-ordinated rhythmic events in crustaceans, possibly including ecdysis: further studies are now timely.

## Ethics Statement

This study involved the use of invertebrates, and thus was not subject to UK Home Office licensing requirements.

## Author Contributions

SW, JA, AO, and DW designed and performed the research. SW, JA, and DW wrote the paper.

## Conflict of Interest Statement

The authors declare that the research was conducted in the absence of any commercial or financial relationships that could be construed as a potential conflict of interest.

## Footnotes

1 http://web.expasy.org/translate/

2 http://www.cbs.dtu.dk/services/TMHMM/

## Abbreviations

*Ag*: abdominal ganglion
BSA: bovine serum albumin
BURS: bursicon
cAMP: 3′-5′cyclic adenosine monophosphate
CCAP: crustacean cardioactive peptide
*cg*, cardiac ganglion, CHO: Chinese hamster ovary
CLR: calcitonin-like receptor
CNS: central nervous system
*coc*, circum- oesophageal ganglion, *cog*, commissural ganglion, CRF: corticotropin releasing factor
CRZR: corazonin receptor
DH31: diuretic hormone 31
DH31R: diuretic hormone 31 receptor
DMEM: Dulbecco’s modified eagle medium
EDTA: ethylenediamine-tetraacetic acid
HEPES: *N*-(2-Hydroxyethyl)piperazine-N′-(2-ethanesulfonic acid)
HPLC: high performance liquid chromatography
IHC: immunohistochemistry
ISH: *in situ* hybridization
*Mda*: *musculus dorsoventralis anterior*
*Mdp*: *musculus dorsoventralis posterior*
MT: Malpighian tubule
*Og*, oesophageal ganglion, ORF: open reading frame
PBS: phosphate buffered saline
PDFR: pigment dispersing factor receptor
PDH: pigment dispersing hormone
PFA: paraformaldehyde
*Po*, pericardial organ, RACE: rapid amplification of complementary ends
RAMP: receptor activity-modifying protein
RCP: receptor component protein
RPCHR: red pigment concentrating hormone receptor
*Stg*, stomatogastric ganglion, Stn: stomatogastric nerve
TFA: trifluoroacetic acid
TR-FIA: time-resolved fluoroimmunoassay.
