# Supplementary material for: Functional Identification and Characterization of the Diuretic Hormone 31 (DH31) Signaling System in the Green Shore Crab, Carcinus maenas
Source: Front Neurosci. 2018 Jul 4;12:454. doi: 10.3389/fnins.2018.00454 (PMC6039563; doi:10.3389/fnins.2018.00454)
Supplement: Supplementary file 1 [file Table_1.DOCX]

**SUPPLEMENTARY TABLES Table 1. Primer and probe sequences for PCR, quantitative Taqman PCR and in situ hybridization**. Each Taqman assay includes a forward and reverse primer (F’ and R’) and a hydrolysis probe labeled at the 5’ end with a fluorescent dye and a minor groove binding 3’ end. ‘Standard synthesis’ refers to primer pairs used in the generation of PCR templates or for *in vitro* transcription of qPCR standard curves. The T7 phage promoter site (T7) used had the sequence 5’-TAATACGACTCACTATAGGG-3’

.

| Method | Oligonucleotide name | Sequence (5’-3’) |
| --- | --- | --- |
|  |  |  |
| *DH-31* Taqman qPCR assay | Cam DH-31 Taqman probe | NED-CGTCCTCCTATCCTC -MGB |
|  | Cam DH-31 Taqman F’ | TCGTTACCCTGGTGGCTACTG |
| *DH-31R* Taqman qPCR assay | Cam DH-31 Taqman R’  Cam DH-31R Taqman probe  Cam DH-31R Taqman F’  Cam DH-31R Taqman R’ | TGTTGAGTGGAGAAGCGTTGA  NED-CCCTCGAACCTTTG  TCCTTCCCTGGGTGCTACT  TTCCAACAGGCCATCCA |
|  |  |  |
|  |  |  |
|  |  |  |
| *UbcE2L3* Taqman qPCR assay | CamUbcE2 Taqman probe | FAM-ACCCGAGAACCCACC-MGB |
|  | CamUbcE2 Taqman F’ | TCACCTGGCAGGGACTCATT |
|  | CamUbcE2 Taqman R’ | CCTGAACGCTCCCTTGTTGT |
| *Elf1a* Taqman qPCR assay | CamElf1a Taqman probe | VIC-CTCTCTTTGACGCTCTGG-MGB |
|  | CamELF1a Taqman F’ | GAGCGGCAGCTATGAGTTCAT |
|  | CamElf1a Taqman R’ | TGGATGGAGGCTCAATGTTG |
|  |  |  |
| *DH-31* standard synthesis | Cam DH-31 STD T7 F’ | T7-CTCGACGTGGAGGAAAACAT |
|  | Cam DH-31 STD T7 R’ | T7-AGGAAGTCCTTCGCTTGTGA |
|  |  |  |
| *DH-31R* standard synthesis | Cam DH-31R STD T7 F’  Cam DH-31R STD R’ | T7-CAACAGCAGACGTGGATTTG  TTGGTTTGTGGGTGTCTGAA |
| *Elf1a* standard synthesis | CamElf1a STD T7 F’ | T7-CCAAGATCGAGCGTAAGAGC |
|  | CamElf1a STD R’ | CGATCACCTGAGCTGTGAAA |
| *UbcE2L3* standard synthesis | Cam UbcE2 STD T7 F’ | T7-AGTCGTTCCGGGACATACAG |
|  | Cam UbcE2 STD T7 R’ | T7-GGCCTCTTCTCGGAGTTCTT |
|  |  |  |
| DH-31R directional cloning | DH-31R 68 F’ CACC | CACCGTGATGGAGGGCAACTCA |
|  | DH-31 R 1396 R’ | TTACACGTCATCACCCTCGATAGTTG |
