# Supplementary material for: Functional Identification and Characterization of the Diuretic Hormone 31 (DH31) Signaling System in the Green Shore Crab, Carcinus maenas
Source: Front Neurosci. 2018 Jul 4;12:454. doi: 10.3389/fnins.2018.00454 (PMC6039563; doi:10.3389/fnins.2018.00454)
Supplement: Supplementary file 2 [file Table_2.DOCX]

**SUPPLEMENTARY TABLES Table 2: Peptides used in receptor assays.** Abbreviations: A-C5-M; Alexa 488-C5-maleimide-C, CRF; corticotropin releasing factor-like, PDH-1; pigment dispersing hormone-1.

*Carcinus maenas* DH31 GLDMGLGRGFSGSQAAKHLMGLAAANYAGGP-NH_2_

*Diploptera punctata* DH31 GLDLGLSRGFSGSQAAKHLMGLAAANYAGGP-NH_2_

*Tribolium castaneum* DH31 GLDLGLGRGFSGSQAAKHLMGLAAANFAGGP-NH_2_

*Drosophila melanogaster* A-C5-M-C DH31 TVDFGLARGYSGTQEAKHRMGLAAANFAGGP-NH_2_

*Rhodnius prolixus* CRF MQRPQGPSLSVANPIEVLRSRLLLEIARRRMKEQDASRVSKNRQYLQQIG

*Carcinus maenas* PDH-1 NSELINSLGLPKVMNDA-NH_2_
