# Supplementary material for: Functional Identification and Characterization of the Diuretic Hormone 31 (DH31) Signaling System in the Green Shore Crab, Carcinus maenas
Source: Front Neurosci. 2018 Jul 4;12:454. doi: 10.3389/fnins.2018.00454 (PMC6039563; doi:10.3389/fnins.2018.00454)
Supplement: Supplementary file 3 [file Image_1.PDF]

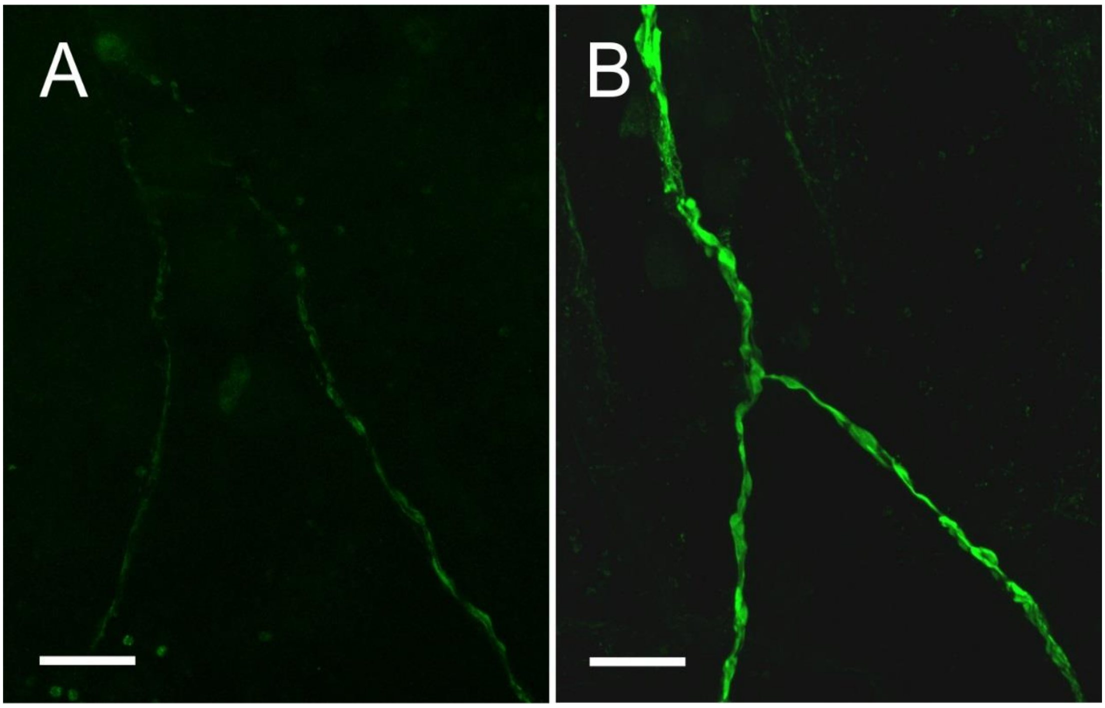

SUPPLEMENTARY FIGURE 1 Preabsorbtion controls: Affinity purified DH31 1/10,000 was applied to ventral ganglia, and processed for IHC as detailed in Materials and Methods. Preabsorbtion of antiserum was performed with equimolar quantities of DH 31, co-incubated with antiserum (1:10 dilution) for 24h, 4ºC before final dilution. Confocal microscopy was performed at maximum signal amplification for the preabsorbtion controls, showing almost complete removal of signal A), compared to B) preparations of the same area of the CNS (the sub-oesophageal ganglion axon fascicles) using exactly the same procedure, but without hormone, at normal amplification settings. Scale bars, 200μm.
